# Osteosarcoma in the distal femur two years after an ipsilateral femoral shaft fracture: a case report

**DOI:** 10.1186/1752-1947-5-198

**Published:** 2011-05-21

**Authors:** Oscar Dorrestijn, Paul C Jutte

**Affiliations:** 1 Department of Orthopedic Surgery, University Medical Center Groningen, University of Groningen, PO Box 30.001, NL-9700 RB Groningen, The Netherlands

## Abstract

**Introduction:**

The duration of symptoms preceding a definitive diagnosis of osteosarcoma is quite long. Pathological radiological signs are often evident by the time of diagnosis. Although several case reports have been published on osteosarcoma of the femur, to the best of our knowledge this report is the first one with such an unusual clinical course.

**Case presentation:**

We describe the case of a 58-year-old Caucasian man who presented with a femoral shaft fracture. Two years post-trauma osteosarcoma in the ipsilateral distal femur was diagnosed. Was it coincidence? We think that the history of the trauma is crucial to answering this question.

**Conclusion:**

This case report underlines the need to keep up awareness of pathological fractures in emergency medicine and trauma surgery. When radiographs do not raise any suspicion but the history of trauma or the physical examination does, we recommend further radiological and/or histological diagnostic examinations.

## Introduction

The "symptom interval" is defined as the time from the first onset of symptoms or signs until a definitive diagnosis is made and treatment is initiated. In people with bone cancer, this interval can be quite long. For patients with osteosarcoma, a median symptom interval of 3.8 months (range 1.0-14.6) is reported for patients between ages 12 and 20 years [1]. Both patient and professional delays contribute to this prolonged delay. A minority of patients with osteosarcoma present with pathological fractures. Guerra *et al*. [2] reported 20 such cases (11.4%) of 175 total patients in their series. The pathological nature of the fracture is commonly recognized on the basis of radiological signs. We report a case of osteosarcoma in the distal femur diagnosed two years after an ipsilateral femoral shaft fracture. Was it a pathological fracture or was it coincidence?

### Case presentation

**Figure 1:**
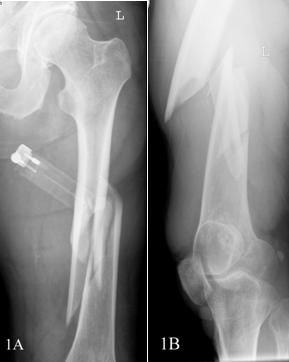
**(A) Anteroposterior view of the patient's left femur. **(B) **Attempt at a lateral view of the left femur**.

**Figure 2:**
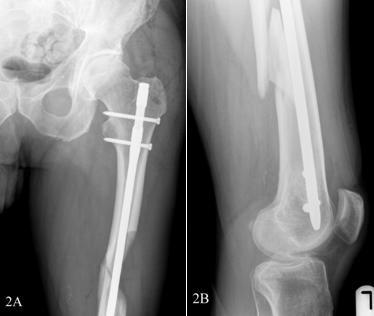
**(A) Anteroposterior view of the proximal femur with the intramedullary nail *in situ*. (B) Lateral view of the distal femur**.

**Figure 3:**
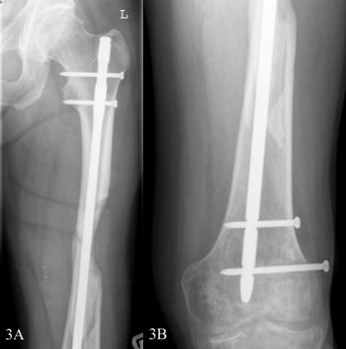
**(A) Anteroposterior view of the proximal femur four months after bone grafting. (B) Migration of the distal screws**.

**Figure 4:**
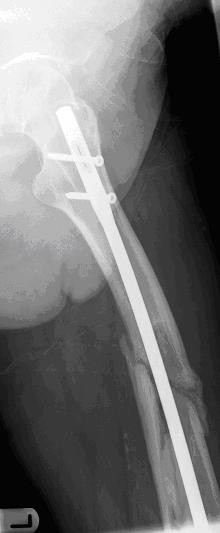
**Some bony callus at the fracture site**.

A 58-year-old, previously healthy Caucasian man visited our emergency department after stumbling in a local pub. He complained of pain in his left upper leg and was not able to bear weight on it. An examination showed a large swelling on his upper leg that was very tender upon palpation, and his leg was shortened. Radiographs showed a comminuted spiral fracture of the femoral shaft, AO type 32 C1.1 (Figures 1A and 1B). A closed reduction of the fracture and an internal fixation with an Unreamed Femoral Nail (UFN; Synthes BV, Zeist, the Netherlands) (Figures 2A and 2B) were performed. Postoperative radiographs showed a persistent marked diastasis between the fracture fragments. One large fragment was shifted dorsomedially. After a six-month period, no signs of consolidation were seen. The patient complained of pain at the level of the fracture as well as at the distal femur, just above the knee. A second operation was performed for autologous bone grafting with bone harvested from the iliac crest. The patient's weight-bearing was increased, but consolidation of the fracture did not progress. Ten months post-trauma there was still no callus at the fracture site, and the patient's pain at the level of the distal femur persisted (Figure 3A). The pain was thought to be caused by the migrating distal screws (Figure 3B); therefore, the screws were removed. The osteolytic area in the distal femoral metaphysis was explained as bone loss resulting from immobilization. Thirteen months postoperatively some bony callus appeared (Figure 4), and the patient increased weight-bearing without crutches.

**Figure 5:**
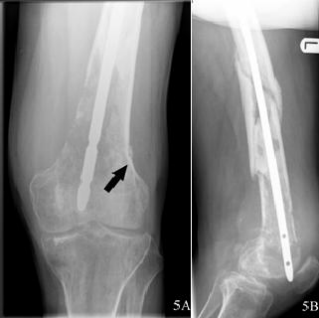
**(A) Osteolytic lesion in the distal femur and a fracture line (arrow). (B) Besides the osteolytic lesion in the distal femur, lytic areas are visible at the fracture level**.

Two years post-trauma the patient visited our first-aid department again. Over the previous five weeks, there had been progressive pain in his left knee, which had suddenly become exacerbated during the previous week after a misstep. Since that moment, he had been unable to bear weight on it. A physical examination showed a swollen left knee, and all movements of the knee were extremely painful for the patient. Laboratory data revealed no abnormalities. The radiographs (Figure 5A), however, showed an osteolytic lesion in the distal femur and a fracture line at the level of the proximal aspect of the lateral femoral condyle (Figure 5A, arrow). That was the first moment during follow-up when the suspicion of a malignancy arose. Sequential computed tomographic scans of the thorax and triple-phase, whole-body bone scintigraphy did not reveal signs of metastases. The bone scan showed an increased uptake in the left lateral femoral condyle and less intensively in the midshaft. Reduced uptake was seen at the left medial femoral condyle. A Jamshidi needle biopsy was performed from the most suspicious region at the medial femoral condyle. No biopsy was performed at the fracture site. The histopathological examination showed an undifferentiated lytic lesion matching a pleomorphic osteosarcoma.

An exarticulation of the hip followed. Prior to the operation, the last radiographs were taken (Figure 5B). The histology showed a classic high-grade osteosarcoma (Enneking rating stage IIB). At the last follow-up examination, three years after undergoing amputation, the patient was alive without metastatic disease and was able to ambulate with crutches.

## Discussion

We present a rare case of a patient with osteosarcoma in the distal femur diagnosed two years after a fracture in the femoral shaft. In our patient, there were no clues of the pathological nature of the fracture; therefore, neither additional imaging studies nor a biopsy was performed initially. Retrospectively speaking, we did not see any signs of malignancy on the trauma radiographs either. Besides, most osteosarcomas originate in the metaphyseal region as mixed sclerotic and lytic lesions, although primarily sclerotic or lytic lesions also occur. Most osteosarcomas appear with destruction of the bony cortex and the formation of a soft-tissue mass [3].

In this case, the question is whether the presence of the femoral shaft fracture and the osteosarcoma in the same femur were coincidental. We do not think they were. Although no biopsy was performed at the fracture site, we believe the femoral shaft fracture had a pathological nature. Several facts speak for this. The patient stumbled in a local pub. Such a traumatic mechanism is unlikely to cause the femoral shaft fracture presented; therefore, we think a primary tumor existed at the site of the fracture and was spread distally by the insertion of the intramedullary nail. The initial trauma imaging studies did not reveal any signs of a pathological fracture, but the last radiographs (Figure 5B) showed lytic lesions in different fracture fragments. According to our hypothesis, the osteosarcoma in the distal femur would be an iatrogenic skip metastasis. Skip metastases are defined by the American Joint Committee on Cancer as "two or more discontinuous lesions in the same bone" [4]. Another case of iatrogenic spread of bony metastasis was described by Currall *et al*. [5]. They presented a case of synovial metastases with bony metastasis following total knee arthroplasty.

Regarding skip metastases, it is known that they tend to be less differentiated than the primary tumor [6]. In our case, the histology of the distal femur biopsy showed an undifferentiated lesion. This finding supports our hypothesis that the lesion in the distal femur would be the skip lesion.

A prolonged union time of 13 months, despite bone grafting, is also quite unusual, especially in a case of intramedullary nailing. The mean reported union time for femoral shaft fractures caused by a fall is 11.1 weeks, and it is 10.4 weeks for intramedularry nailing [7]. Even after bone grafting, the first bony callus was seen only seven months after pseudarthrosis repair in our patient. This observation might also speak for a pathologic fracture. Another option is that the lytic lesions at the fracture were skip metastases from a primary osteosarcoma in the distal femur; therefore, the appearance of a femoral shaft fracture and an osteosarcoma in the same femur could be coincidental.

Antegrade femoral nailing is the gold standard for surgical treatment of diaphyseal femoral shaft fractures because of decreased operative trauma and the possibility of early weight bearing [8]. It has a high rate of union (99%) and a low rate of infection and malunion (<1%) [9,10]. One of the complications in the surgical treatment of femoral shaft fractures is delayed union or nonunion. The nonunion in our case could be a result of the marked diastasis of the fracture fragments.

Facts supporting the suspicion of a primary tumor in the distal femur are the location and size of the lesion. The distal femur is a far more frequently affected site compared to the diaphyseal femur: 44% and 3%, respectively [2]. In terms of size, the lesion in the distal femur of our patient had progressed much more than the lesions in the fracture fragments (Figure 5B). Another argument is that skip metastases in osteosarcomas are more often found proximal to the primary tumor [6].

## Conclusion

Although we do not have histological proof for the femoral shaft fracture being pathological in our patient, we think the answer must be sought in the history of the trauma that caused this fracture. The absence of signs of malignancy on the first plain film radiographs and the later development of the osteosarcoma emphasize the importance of a detailed history of the trauma mechanism. An inadequate trauma mechanism can be an important clue for the pathological nature of a fracture.

This case report underlines the need to keep up awareness of pathological fractures in emergency medicine and trauma surgery. When radiographs do not raise any suspicion but the history of trauma or the physical examination do, we recommend further radiological and/or histological diagnostic examinations.

## Consent

Written informed consent was obtained from the patient for publication of this case report and accompanying images. A copy of the written consent is available for review by the Editor-in-Chief of this journal.

## Competing interests

The authors declare that they have no competing interests.

## Authors' contributions

PCJ provided the concept and clinical data for this case report. OD was the major contributor in writing the manuscript. Both authors read and approved the final manuscript.
